# Diagnostic performance of urinary IgG antibody detection: A novel approach for population screening of strongyloidiasis

**DOI:** 10.1371/journal.pone.0192598

**Published:** 2018-07-09

**Authors:** Chatanun Eamudomkarn, Paiboon Sithithaworn, Christine Kamamia, Anna Yakovleva, Jiraporn Sithithaworn, Sasithorn Kaewkes, Anchalee Techasen, Watcharin Loilome, Puangrat Yongvanit, Chompunoot Wangboon, Prasert Saichua, Makoto Itoh, Jeffrey M. Bethony

**Affiliations:** 1 Department of Parasitology, Faculty of Medicine, Khon Kaen University, Khon Kaen, Thailand; 2 Cholangiocarcinoma Research Institute (CARI), Khon Kaen University, Khon Kaen, Thailand; 3 Department of Microbiology, Immunology & Tropical Medicine, George Washington University, Washington, D.C., United States of America; 4 Faculty of Associated Medical Sciences, Khon Kaen University, Khon Kaen, Thailand; 5 Faculty of Medicine, Mahasarakham University, Mahasarakham, Thailand; 6 Department of Biochemistry, Faculty of Medicine, Khon Kaen University, Khon Kaen, Thailand; 7 Biomedical Science Program, Graduate School, Khon Kaen University, Khon Kaen, Thailand; 8 Tropical Medicine Program, Faculty of Medicine, Khon Kaen University, Khon Kaen, Thailand; 9 Department of Infection and Immunology, Aichi Medical University School of Medicine, Nagakute, Aichi, Japan; National Institutes of Health, UNITED STATES

## Abstract

The diagnosis of strongyloidiasis by coprological methods has a low sensitivity, underestimating the prevalence of *Strongyloides stercoralis* in endemic areas. Serodiagnostic tests for strongyloidiasis have shown robust diagnostic properties. However, these methods require a blood draw, an invasive and labor-intensive sample collection method, especially in the resource-limited settings where *S*. *stercoralis* is endemic. Our study examines a urine-based assay for strongyloidiasis and compares its diagnostic accuracy with coprological and serological methods. Receiver operating characteristic (ROC) curve analyses determined the diagnostic sensitivity (D-Sn) and specificity (D-Sp) of the urine ELISA, as well as estimates its positive predictive value and diagnostic risk. The likelihood ratios of obtaining a positive test result (LR+) or a negative test result (LR-) were calculated for each diagnostic positivity threshold. The urine ELISA assay correlated significantly with the serological ELISA assay for strongyloidiasis, with a D-Sn of 92.7% and a D-Sp of 40.7%, when compared to coprological methods. Moreover, the urine ELISA IgG test had a detection rate of 69%, which far exceeds the coprological method (28%). The likelihood of a positive diagnosis of strongyloidiasis by the urine ELISA IgG test increased significantly with increasing units of IgG detected in urine. The urine ELISA IgG assay for strongyloidiasis assay has a diagnostic accuracy comparable to serological assay, both of which are more sensitive than coprological methods. Since the collection of urine is easy and non-invasive, the urine ELISA IgG assay for strongyloidiasis could be used to screen populations at risk for strongyloidiasis in *S*. *stercoralis* endemic areas.

## Introduction

Strongyloidiasis is a neglected tropical disease (NTD), with transmission occurring in tropical and subtropical regions of the world, including the subtropical regions of the United States (Southeastern USA) [1–3]. People acquire an infection via penetration of the skin by infective larvae whereupon the larvae enter the blood circulation, reaching the lungs and subsequently the gastrointestinal tract where they mature to adult worms. The life cycle of *S*. *stercoralis*, however, is unique among soil-transmitted helminths (STHs) in several key respects. *Strongyloides stercoralis* filariform larvae can autoinfect its human host by re-entering via enteral circulation without shedding larvae into the soil. With both irregular and minimal *S*. *stercoralis* larval output in human feces, conventional microscopic methods often fail to detect chronic asymptomatic strongyloidiasis. Despite the regular daily collection of stool samples which were also subjected to fecal concentration techniques, coprological tests by the Baermann and Koga agar plate culture (ACP) have been found to lack significant diagnostic sensitivity (4). Hence, improved methods for the detection of *S*. *stercoralis* infection are urgently needed not only for people at increased risk from chronic strongyloidiasis (e.g., candidates for transplantation, people undertaking chemotherapy, or people on systemic corticosteroids) [1,4], but also people residing in *S*. *stercoralis* endemic areas, such as northeast Thailand, where the current study takes place.

Several serological tests to detect chronic strongyloidiasis have been developed, resulting in dramatically increased diagnostic sensitivity [5]. These serum or plasma based indirect enzyme-linked immunosorbent assays (ELISA) is often based on a crude extract of larval antigen of *S*. *stercoralis*, or heterologous antigen from other *Strongyloides* spp. or recombinant *S*. *stercoralis* antigens [5–12]. In a recent evaluation of the diagnostic accuracy of five different serological methods to detect *S*. *stercoralis*, Bisoffi and colleagues, showed that these methods had much greater sensitivity (D-Sn) and specificity (D-Sp) compared to the composite reference method. Hence, they can now act as first line detection methods, especially for individuals awaiting transplantation or immune therapy [5].

However, serodiagnosis requires a blood draw, which is an invasive procedure and not always possible in the resource-poor settings where *S*. *stercoralis* is endemic. Other clinical specimens such as urine or saliva, which can be easily collected would be preferable sample matrices for diagnosis and screening of strongyloidiasis. The detection of antibodies in urine has been suggested as a possible non-invasive alternative technique to diagnose various other diseases such as rubella, hepatitis A and C, *Helicobacter pylori* infection, echinococcosis, leishmaniasis, filariasis, schistosomiasis and opisthorchiasis [13–20]. In addition to antibody detection, DNA-based detection methods in feces or urine have been reported with better diagnostic accuracy than conventional fecal examination techniques [21,22]. However, to date, there have been no reports examining the usefulness of urine specimens for the immunodiagnostis of strongyloidiasis.

We developed and evaluated, herein, a novel urine based indirect ELISA for the diagnosis of strongyloidiasis by the detection of *Strongyloides*-specific immunoglobulin G (IgG) antibody using *Strongyloides ratti* antigen. We have evaluated the diagnostic performance of urine ELISA for *Strongyloides* by comparing it to the conventional coprological methods and the recently developed serological methods in the sample set of urine, fecal, and blood specimens collected simultaneously from individual resident in an *S*. *stercoralis* endemic area in northeast Thailand. Our urine-based detection method is intended for use in endemic settings (to screen people at risk for complications, in prevalence studies, and clinical diagnosis in adequately equipped laboratories), and in areas of low or no endemicity (screening and diagnosis of immigrants, travelers, and autochthonous infection in elderly patients in countries previously endemic, such as in Southern Europe).

## Materials and methods

### Study area and sample collection

**Fig 1:**
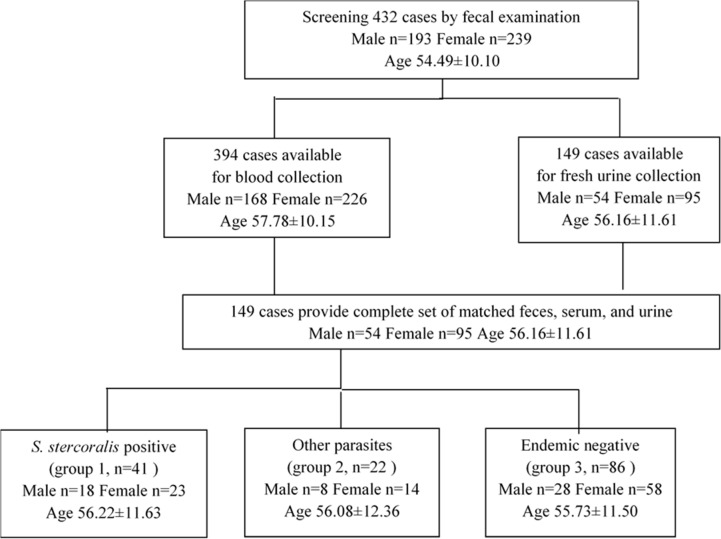
Flow chart of participants in the study. Data shown for age are mean ± SD, where n is the sample size.

We conducted a prospective cross-sectional study from January to April 2010 by surveying individuals by households in the Muang (or city) district, Khon Kaen Province, northeast Thailand, where *S*. *stercoralis* is endemic. Individuals aged 22–86 years of age (inclusive) were recruited for the study, with 149 individuals providing a complete set of samples: feces, blood, and urine (Fig 1). Fecal specimens were kept in a chilled insulated box (at approximately 15°C) and transported to the laboratory at the Khon Kaen University Hospital. A ten milliliter venous whole blood draw was allowed to clot at room temperature for 30 min and sera were separated into aliquots and stored at -20°C. A morning urine specimen was collected in wide mouth containers, centrifuged at 1,500 rpm at 4°C for 15 min, and the supernatant separated with a final concentration of 0.1% NaN_3_ which was added as a preservative [15]. The urine specimens were stored at 4°C until used. The participants were assigned to 3 groups based on the results of fecal examinations. Group 1 was *S*. *stercoralis*-positive (n = 41), Group 2 was other parasitic infections but negative for *S*. *stercoralis* (n = 22). Group 3 was parasite-negative (n = 86) as confirmed by FECT and APCT. These 3 groups of subjects were defined as “field-collected samples”, with no known history of treatment with anthelmintic drugs as determined by interview and Ministry of Health records of the area. A separate set of participants from known non-endemic areas of *S*. *stercoralis* were included to establish the negative controls for the urine ELISA. The parasite infection status was confirmed by fecal examinations (FECT and APCT). There were 75 subjects from whom serum samples were obtained, and 62 subjects who provided urine samples.

### Blood examinations

Blood samples from 121 out of 149 subjects from the field collected samples (Group 1, 2 and 3) were processed for indirect eosinophil counts using a complete blood count (CBC) analyses. The number of absolute blood eosinophils was used to assess eosinophilia (eosinophil > 500 cell/μl) [23]. The CBCs were analyzed by an automated hematological machine (Sysmex KX-21TM-Hematology-Analyzer, Japan).

### Fecal examinations

Fecal examinations were carried out using the agar plate culture technique (APCT) and the quantitative formalin-ethyl acetate concentration technique (FECT). The APCT was performed according to the method described by Koga and coworkers [24]. In brief, four grams of fecal sample was placed on a 1.5% nutrient agar plate and incubated at 25°C for 4–5 days. For parasite identification, the surface of the plate was washed with 10 ml of 10% formalin, transferred to a test tube, centrifuged and the sediment was examined as a wet preparation under a light microscope. In addition, 2 g of fecal sample from each sample was processed for parasitic examination by FECT [10,25]. In FECT, results from duplicate examination of each fecal sample were combined. The sample was defined as positive if at least one *S*. *stercoralis* larvae was found by either method. The intensity of parasitic infection was estimated by egg per gram of feces (epg) and larva per gram of feces (lpg) from FECT.

### Preparation of crude *S*. *ratti* antigen extract

The life cycle of *S*. *ratti* has been maintained in Wistar rats at the Department of Infection and Immunology, Aichi Medical University School of Medicine, Japan. Feces of infected Wistar rats were cultured using a filter paper culture method [26] to produce third-stage filariform larvae (L3) of *S*. *ratti*. The larvae were concentrated and washed with normal saline and stored at -20°C for crude soluble antigen extraction. Antigens of *S*. *ratti* were prepared as described previously [27,28]. *S*. *ratti* L3 were dispersed in phosphate buffer saline (PBS) pH 7.4 containing protease inhibitor mix (GE Healthcare, Bio-Sciences Corp., USA) and were frozen at -70°C for 30 minutes and thawed for 4–5 times and subsequently disrupted by sonication. The homogenate was stored at 4°C overnight and centrifuged at 15,000xg for 30 minutes at 4°C. The protein concentration of the supernatant was measured by the Bradford protein assay [29], then stored at -20°C until used.

### Procedures for urine and serum ELISA

Establishment of the ELISA protocols were conducted based on modifications from the previous studies by our group [10,30]. For the serological studies, antibody levels were expressed as units based on a standard curve made from serially diluted pools of high-titer positive sera from strongyloidiasis patients. A set of the serially diluted sera was included in each microtiter plate in duplicate. Furthermore, eight wells per ELISA set were assigned as internal controls consisting of two blanks, and positive and negative controls in duplicate. Pools of high-antibody titer sera from strongyloidiasis cases were used as the positive control sera to optimize the protocol for urine ELISA.

For the urine ELISA, optimum dilutions of coating antigen, urine samples, anti-human immunoglobulin HRP conjugate and standard curves were predetermined by checkerboard titration. Based on a preliminary study, the urine samples were preserved with a final concentration of 0.1% NaN_3_ and kept at 4°C until required for analyses.

For the standardized ELISA procedure, a 96-well microtiter plate (Maxisorp; Nunc, Roskilde, Denmark) was coated with 5 μg of *S*. *ratti* antigen/ml kept at 4°C overnight. The plates were washed twice with PBS (pH 7.2, containing 0.05% Tween20). After blocking the plate with blocking buffer containing 3% skimmed milk in PBS and 0.5% Tween 20 for 2 hours at room temperature, 100 μl of 8,000 times diluted serum or 100 μl of 2 times diluted urine were added to the wells and incubated for one hour at 37°C. After washing the plates three times, 100 μl of horseradish peroxidase conjugate goat anti-human IgG (dilution 1:4000) (Zymed, California, USA.) was added and incubated at 37°C for 1 hour. The plate was washed three times and 100 μl of a substrate solution (*o*-phenylenediamine in citrate phosphate buffer, pH 5.0) was added and incubated at room temperature in the dark for 1 hour. The enzyme reaction was stopped with 50 μl per well of 4N sulfuric acid and the optical density (OD) of each well was measured at 492 nm by an ELISA reader (TECAN Sunrise, Austria). Each sample was added to the wells in duplicate.

### Cross reactivity

In order to assess the specificity of the urine ELISA for diagnosis of strongyloidiasis, cross reactivity with other parasitic infections and related diseases were investigated. Urine samples from subjects with other parasitic infections were tested: *Opisthorchis viverrini* (n = 15), *Taenia* sp. (n = 7), *Trichuris trichiura* (n = 4), *Echinostoma* sp. (n = 6), minute intestinal flukes (n = 8). In addition, urine from other diseases available for testing included cholangiocarcinoma (n = 4), cholecystitis (n = 3), and adenocarcinoma of different organs (n = 12), with 5 from the rectum, 2 from the colon, 2 from the pancreas, 1 each from the stomach, gall bladder and liver. Cross reactivity analyses with serum included *O*. *viverrini* (n = 17), *Taenia* sp. (n = 2), *Angiostrongylus cantonensis* (n = 2), *Paragonimus* spp. (n = 4), *Fasciola* spp. (n = 5) and *Clonorchis sinensis* (n = 5). The procedures for collection of urine and serum samples for cross reactivity tests were the same as those for field-collected samples.

### Blinding

Laboratory staff involved in the study had no access to the urine and serum codes or the clinical information of participants, therefore the results of the references tests and the index tests were blinded during the specimen analyses.

### Statistical analysis

A combination of parasitological techniques of APCT and FECT were used as the primary reference standard. Due to a low sensitivity of the gold standard parasitological techniques for *S*. *stercoralis*, a composite reference standard was used in this study as previously suggested [5]. The composite reference standard to calculate the diagnostic accuracy for serum ELISA was a combination of the results of parasitological diagnoses (fecal examination) and urine ELISA. In the case of urine ELISA, the composite reference standard was a combination of the results of parasitological diagnoses and serum ELISA.

Receiver Operating Characteristic (ROC) curves were used to evaluate the diagnostic parameters of the urine ELISA and serum ELISA compared to the primary and composite reference standard. The ROC curve was used to calculate the sensitivity, specificity, positive predictive values (PPV), negative predictive values (NPV) the likelihood ratio of obtaining a positive test result (LR+), and the likelihood ratio of obtaining a negative test result (LR-) using a 50% *S*. *stercoralis* prevalence rate, as previously determined from our field studies in the *S*. *stercoralis* endemic areas in the region [27] to further characterize assay performance.

A logistic regression model was used to evaluate the relationship between strongyloidiasis infection status and urine and serum antibody concentrations determined by the urine ELISA and serum ELISA assays. The model was used to calculate odds ratios (OR) and corresponding 95% confidence intervals (CIs). A 0.05 significance level (alpha = 0.05) was utilized to determine meaningful predictors in the model. Pearson product-moment correlation test was used to evaluate the correlation coefficient between urine and serum antibodies and the correlation between blood eosinophil count and urine or serum antibodies. Statistical analyses were performed with SPSS version 21 (IBM) and SAS 9.3 (Cary Institute, NC).

### Ethics statement

The study protocol was approved by the Ethics Committee of Khon Kaen University, Khon Kaen Thailand (reference number HE561057). Written informed consents were obtained from all participating subjects. Infected individuals were treated with appropriated anthelmintic drugs. The protocol for the maintenance and production of larval stages of *S*. *ratti* was approved by the Institutional Animal Ethics Committee of the Aichi Medical University (reference number 2013–17).

## Results

### Study sample

There were 149 individuals (54 males and 95 females with an average age of 54 years) who provided a complete set of urine, feces and blood samples in this study (Table 1).

**Table 1:** Demographic data and detail parasitic infections of the field collected sample subjects for assessment of diagnosis performance study. Data shown were number of subjects, mean and S.D.

Group	*S*. *stercoralis* monoinfection (Group 1)	Other parasite infection (Group 2)	Negative (Group 3)	Total
Total	41	22	86	149
Male	18	8	28	54
Female	23	14	58	95
Age (years) (Mean ±SD)	57.2 ± 12.1	59.6 ±13.1	54.9±10.9	56.2 ± 11.6
Age by strata				
20–40	2	1	3	6
41–60	20	10	59	89
60+	19	11	24	54
Intensity of Infection				
LPG*	18.2 ± 9.2	0	0	

*LPG refers to larvae per gram of feces

### Comparative diagnostic accuracy of urine, fecal and serum detection methods

**Fig 2:**
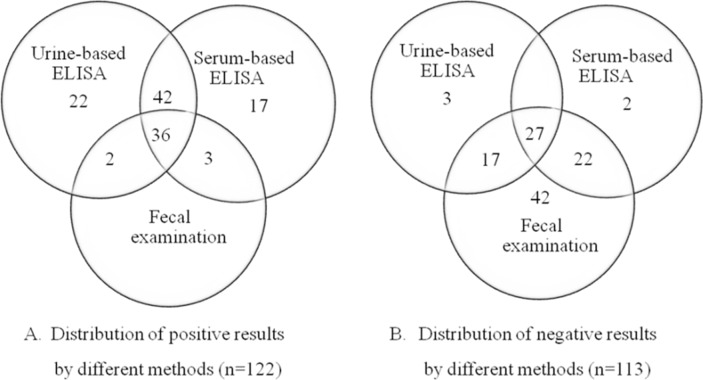
Venn diagrams comparing the distribution of positive and negative results by each diagnostic method.

The Venn diagrams (Fig 2) show the overlapping distributions of strongyloidiasis positive and negative results for each detection method. Among 122 cases (81.9%) (Fig 2A), 78 cases (52.3%) were positive by both urine and serum ELISA, whereas 22 cases (14.8%) were positive only by urine ELISA and 17 cases (11.4%) positive by serum ELISA. Of the 102 positive cases by urine ELISA examination, 4 cases (3.9%) were found to be negative by serum ELISA. When the positive results from the two ELISA methods and the fecal examination were analyzed using Venn diagram, 36 cases were positive for all three methods (24.2%), 2 were positive for urine ELISA and fecal examination and 3 were positive by serum ELISA and fecal examination. Conversely (Fig 2B), among a total of 113 negative results, 27 were negative for all three methods. Of those negative results, 42 cases were found to be exclusively negative for fecal examination.

### Cross-reactivity with other parasitic infections endemic to Northeastern Thailand

**Fig 3:**
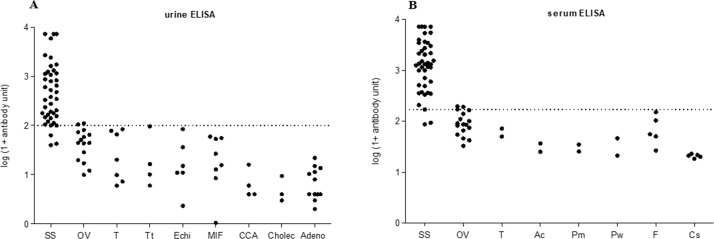
Tests for cross reactivity with other parasites and disease for strongyloidiasis. Tests for cross reactivity of the urine ELISA (A) and serum ELISA (B) for strongyloidiasis. (SS, *S*. *stercoralis*; OV; *O*. *viverrini*; T, *Taenia* sp.; Tt, *T*. *trichuira*; Echi, Echinostomes; MIF, minute intestinal flukes; Ac, *A*. *cantonensis*; Pw, *P*. *westernmani*; Pm, *P*. *miyazakii*; F, *Fasciola* spp.; Cs, *C*. *sinensis*; CCA, cholangiocarcinoma, Cholec, cholecystitis; and Adeno, adenocarcinoma).

**Fig 4:**
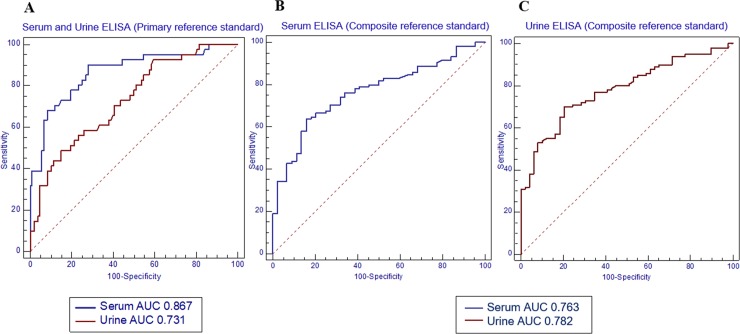
The comparison of the ROC curves. The ROC curve illustrates the comparison between the diagnostic performance of antibody detection using a urine assay and a serum assay. The model used to construct the ROC curve was modeled to include negative controls (strongyloidiasis negative and other infections) and individuals who were infected with strongyloidiasis. A; Primary reference standard for serum and urine ELISA, B; Composite reference standard for serum ELISA, C; Composite reference standard for urine ELISA.

Apart from the field-collected samples, separate sets of urine and serum samples were tested for cross reactivity of the urine and serum ELISA for an evaluation of specificity. As shown in Fig 3, no cross-reaction for urine ELISA was found in samples from individuals infected with *Taenia* sp. (n = 7), *T*. *trichiura* (n = 4), *Echinostoma* sp. (n = 6), minute intestinal flukes (n = 8), cholangiocarcinoma (n = 4), cholecystitis (n = 3), and adenocarcinoma (n = 12). For serum ELISA, there was no cross-reaction from individuals infected with *Taenia* sp. (n = 2), *A*. *cantonensis* (n = 2), *Paragonimus* spp. (n = 4), *Fasciola* spp. (n = 5), and *Clonorchis sinensis* (n = 5). There were cross-reactivity in individuals with *O*. *viverrini* for both urine (2/15) and serum (3/17) ELISA, however, the antibody level was close to the cutoff point. Table 2 shows the threshold to obtain the diagnostic cutoff for positivity using urine ELISA and serum ELISA as determined by the ROC curve (Fig 4). The positive and negative predictive values (PPV and NPV) and positive and negative likelihood ratios (LR+, LR-) were estimated based on a prevalence of *S*. *stercoralis* of 50%. In comparison to the primary reference standard, the urine ELISA ROC had an AUC of 0.731 with 93% sensitivity and 41% specificity and the serum assay had an AUC of 0.867, 95% sensitivity, 45% specificity. Moreover, when compared to the composite reference standard, the urine assay ROC had an AUC of 0.782 with 80% sensitivity and 55% specificity and the serum assay had an AUC of 0.763 with 77% sensitivity and 61% specificity.

**Table 2:** Diagnostic performance of antibody detection by the urine assay and serum assay compared with the primary and composite reference standard.

	A. Primary reference standard (41 infected, 108 uninfected)					
Diagnostic Method	AUC*	Sensitivity (%)	Specificity (%)	Predictive value (%)		^**†**^LR+	^**†**^LR-
				Positive	Negative		
Urine	0.731	92.7	40.7	37.2	93.6	1.6	0.2
Serum	0.867	95.1	45.3	39.8	96.1	1.7	0.1
	B. Composite reference standard					
Diagnostic Method	AUC	Sensitivity (%)	Specificity (%)	Predictive value (%)		^**†**^LR+	^**†**^LR-
				Positive	Negative		
Urine	0.782	80.0%	55.1%	78.4	57.5	1.8	0.4
Serum	0.763	77.1%	61.4%	82.7	52.9	2.0	0.4

*AUC refers to the area under the Receiver operating characteristic (ROC) curve. Positive Predictive Value (PPV), Negative Predictive Value (NPV) and Likelihood Ratios (LR) were estimated using 50% prevalence rate of strongyloidiasis.

^**†**^ LR+ refers to the likelihood of observing a positive test result in patients with strongyloidiasis, and LR- refers to the likelihood, after subtracting from 1, of observing a negative test result with individuals without strongyloidiasis infection

A logistic regression model was used to determine the odds of having a positive diagnosis of *S*. *stercoralis* based on increasing urine antibody levels and serum antibody levels as presented in Table 3. *S*. *stercoralis* infection level as expressed by larvae per gram (lpg) of feces, age, sex, and blood eosinophil counts were included in the model to assess for confounding, however only blood eosinophil count was found to be a significant confounder only when using urine ELISA assay. The confounding effect of the eosinophil count was stronger as the urine antibody levels increased. A one arbitrary unit increase in urine strongyloidiasis as detected by the urine assay had a less than 1% odds of having strongyloidiasis; However, in increasing antibody units of 100, 500 and 1000 the odds of a positive diagnosis were 4%, 20% and 45% respectively when adjusted for blood eosinophil count. Similar results are observed using the serum assay, however the odds of having a positive strongyloidiasis diagnosis are much higher at 20%, 251% and 632% respectively.

**Table 3:** Odd ratios and 95% confidence intervals for predicting the presence of *Strongyloides stercoralis* infection by the detection of IgG against a crude *S*. *ratti* antibody in urinary or serum.

Predicting strongyloidiasis using urine ELISA assay				
Unit	Odds Ratio	95% CI^a^ (Lower, Upper)	Adjusted^b^ Odds Ratio	95% CI^a^ (Lower, Upper)
1.0	0.998	0.997–0.999	1.002	1.000–1.001
100.0	1.057	1.023–1.109	1.038	1.004–1.085
500.0	1.320	1.120–1.680	1.203	1.020–1.503
1000.0	1.742	1.254–2.821	1.446	1.041–2.260
Predicting strongyloidiasis using serum ELISA assay				
Unit	Odds Ratio	95% CI^a^ (Lower, Upper)	Adjusted^b^ Odds Ratio	95% CI^a^ (Lower, Upper)
1.0	1.002	1.001–1.003	0.998	0.997–0.999
100.0	1.202	1.126–1.301	1.206	1.113–1.333
500.0	2.513	1.428–2.200	2.552	1.706–4.213
1000.0	6.317	1.812–2.723	6.513	2.909–17.748

^a^ CI refers to confidence interval.

^b^ Adjusted for eosinophil count.

### Correlations between antibodies in serum and urine

**Fig 5:**
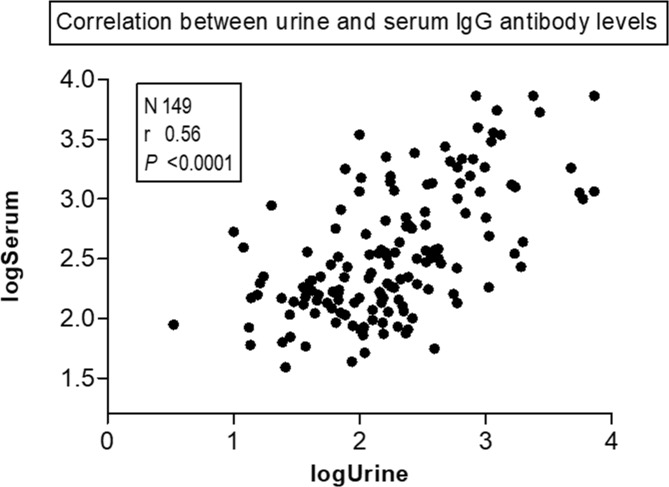
Correlation between urine IgG and serum IgG antibodies. Log-transformed variables. Correlation coefficient determined by Pearson product-moment correlation test.

The correlation of IgG antibody levels obtained by two ELISA methods were analyzed and the results show statistically significant correlations (r = 0.56; *P*<0.0001) between urine and serum antibody levels (Fig 5). Test for agreement between two diagnostic methods revealed fair agreement when compare with primary reference standard and good agreement at composite reference standard as shown in Table 4.

**Table 4:** Test for agreement between two diagnostic methods.

Kappa value				
Diagnostic test	Primary reference standard	Composite reference standard	Serum ELISA	Urine ELISA
Serum ELISA	0.28	0.60	/	
Urine ELISA	0.23	0.65	0.33	/

### Correlation between blood eosinophil count and antibodies in serum and urine

We observed a significant positive correlation between eosinophil and urine antibody (r = 0.25, *P* = 0.006), however, the correlation was relatively weak. The positive correlation between eosinophil and serum antibody was stronger with a correlation coefficient of 0.48 (*P*<0.0001). Table 5 shows the number of cases with eosinophilia separated by diagnostic test results. The strongyloidiasis positive group had higher percentages with eosinophilia than those in the negative group.

**Table 5:** The number of cases with eosinophilia as classified by results of each diagnostic methods. Absolute eosinophil count >500 cell/μl = eosinophilia.

Diagnostic tests	Eosinophilia	
	Positive diagnostic result	Negative diagnostic result
Fecal examination	21/38 (55.3%)	21/83 (25.3%)
Serum ELISA	36/81 (44.4%)	6/40 (15.0%)
Urine ELISA	31/81 (38.3%)	11/40 (27.5%)

## Discussion

The present studies showed that urine and serum ELISA tests for the detection of strongyloidiasis were similar in terms of sensitivity, specificity, and predictive diagnostic values (Odds Ratios). Based on the same sample population, the percent positive for both the urine and serum ELISAs for strongyloidiasis were comparable (68.5% and 65.8%, respectively), while the percent positive for strongyloidiasis by coprological examine was less than half (27.5%) of those for urine and serum tests. In fact, an additional 81 individuals were determined to be positive for strongyloidiasis by urine or serum ELISA that went undetected by the coprological exam. These results show that coprological examination is less sensitive to diagnose strongyloidiasis than urine or serum ELISA. This is in keeping with reports by Siddiqui and Berk [4], who reported that conventional coprological examination fails to detect *S*. *stercoralis* larvae in up to 70% of cases. It should be noted that the detection rate for coprological methods can be increased from 18.6% to 24.4% for *S*. *stercoralis* when repeated fecal samples (usually around 3 days) are combined with the results of APCT and the Baermann method [31]. Moreover, APCT increased sensitivity to 85% when it used with repeated fecal samples [32]. However, three fecal samples are a time-consuming, laborious, and not always a feasible method to screen populations resident in areas where *S stercoralis* is endemic. Our study shows that IgG excreted into urine against a crude larval *S*. *ratti* antigen extract correlated extremely well with IgG level in serum and can also be used to detect strongyloidiasis. By virtue of its increased sensitivity and ease of collecting urine samples, we advise that the urine ELISA for strongyloidiasis be routinely introduced to screen population for strongyloidiasis in *S*. *stercoralis* endemic areas. Chronic strongyloidiasis is often asymptomatic and non-severe, but when left untreated individuals, remain at lifelong risk of hyperinfection syndrome or disseminated strongyloidiasis, both of which can be lethal. Successful identification and treatment of chronic strongylodiasis is therefore critical and this can be accomplished during screening programs of populations [33].

In this study, we showed that the increased detection rate of for strongyloidiasis using the urine and serum ELISA tests resulted in an increased prevalence of *S*. *stercoralis* in an endemic area in northeast Thailand. These findings are consistent with previous studies examining the prevalence of strongyloidiasis using serum ELISA in *S*. *stercoralis* endemic areas in Thailand by Douce and colleagues (45.0%) [7], by Sithithaworn and colleagues (47.5%) [10] and again by Sithithaworn and colleagues (27.5%) [27]. In Malaysia, serum ELISA detection showed that 31.5% prevalence in *S*. *stercoralis* endemic areas [34]. Other studies on prevalence of *S*. *stercoralis* in Southeast Asia based on coprological techniques show lower and much more variable prevalence rates of *Strongyloides*. For example, the prevalence rates were found to be 10.3% in Lao PDR [35], 21.0% in Cambodia [31]; and 21.0% in China [36]. Due to practical reasons, duplicate examinations of a single fecal sample were performed in this study. In addition, false positives for ELISA may occur in light infection cases in which larval output is minimal or may be due to sero-positive reflecting previous *Strongyloides* infection. Moreover, a previous study has shown that in antibody positive cases by ELISA, which had subsequent repeated fecal examinations, approximately half of the cases were found to be infected [32]. For the test of cross reactivity, a previous study using a similar serum ELISA system of *S ratti* showed that there was cross reactivity to other parasites, particularly filariasis [37,38]. Nevertheless, the limitation of this study is that there is no filarial serum samples for cross reactivity. Since, there is no report of filariasis in this study area [39], the possibility of cross reactivity in our test is quite low. The cross reactivity with *O*. *viverrini* is anticipated as concurrent infections by *O*. *viverrini* and *S*. *stercoralis* in northeast Thailand are frequent [10,40]. Another explanation is that *S*. *stercoralis* infection may be in the pre-patent period, therefore the larvae are not detected. The finding that no such cross reactivity for the closely related trematode, *C*. *sinensis* was observed in this study as well as the previous report [27] is currently not known, but confirmed analysis on a larger sample size is required. The diagnostic performance of antibody detection by the urine assay and serum assay compared with the primary reference standard shows 93% and 95% sensitivity respectively, however, PPV are low. When using a combination of diagnostic tests as a standard (composite reference standard), the performances for both serum as well as urine assays had slightly lower sensitivity but had higher specificity and predictive values. The drawback of composite reference standard is that it may cause false positivity, which can be confirmed by parasitological methods.

An important question arising from our study is the presence of detectable *Strongyloides* antibody (protein) in the urine. Theoretically, the permeability of the glomerular barrier should block protein leakage from plasma to urine, such as albumin, which is commonly detected by the urine dipstick test [41]. One explanation for the presence of parasite-specific antibody in urine is that the movement of albumin into Bowman's space is not restricted by pore size, but by its negative charge and the consequent repulsive electrostatic interactions with the negatively charged glomerular endothelium. Hence, it is quite possible that the presence of antibody in the urine due to *S*. *stercoralis* infection is not accompanied by appreciable albuminuria—a hypothesis that clearly deserves further study. However, in normal human urine, immunoglobulins are rarely detectable [42,43] and if so are considered to be associated with renal pathology (microproteinuria). In our study, there were some samples (4 of 102 or 3.9%) that had positive urine ELISA but had negative serum ELISA. We hypothesize that the high concentration of *Strongyloides*-specific IgG detected in the urine, as opposed to low serum concentration, may associate with the degree of deposition of immune complex in the kidneys. This has previously described in schistosomiasis and malaria [44]. Indeed, *Strongyloides*-associated glomerulonephritis as well as the presence of *Strongyloides* antigen in renal biopsy tissues have been demonstrated [45]. Hence, the presence of immune complex in the kidneys as well as vascular inflammation due to glomerulonephritis could enhance leakage of IgG from the plasma to urine which can result in a positive ELISA. Similar to our results, significant correlation between antibody levels in serum and in urine has been reported in the diagnosis of *Schistosoma japonica* and hepatitis-A [15,46]. The correlation between the antibody levels in serum and urine suggest that the antibody in urine is derived from serum. The physiological concentration of IgG in urine is 10,000 times less than that in plasma. Nevertheless, the detection of antibodies in urine have been suggested as the possible alternatives for plasma/serum for the diagnosis of various parasitic diseases because of less invasiveness of the collection of urine samples [13–18]. Collection of urine samples is particularly useful for the epidemiological surveillance when patients are unwilling to give blood samples [47].

In the present study, there was no significant correlation between the antibody level of IgG to crude larval *S*. *ratti* antigen extract and the age, sex or intensity of infection (expressed as larvae per gram of feces or lpg) of the patients, as determined by coprological methods. This result is in concordance with the report from Luvira and coworkers [48]. In contrast to our results, [49–51] there have been reported correlations between antibody levels and age, sex or lpg. This discrepancy maybe due to the insufficient numbers of samples examined in our study or due to the restricted range of sampling in the endemic area.

Although urine sample collection is non-invasive and much easier than serum sample collection, the storage of urine samples can be problematic. Protein concentrations in urine can decrease at -20°C, particularly when precipitates had formed after storage and thawing [52,53]. For urine ELISA for schistosomiasis, Itoh and coworkers [15] reported that antibody levels can be maintained when urine samples were kept at 37°C with 0.1% NaN_3_ for up to 8 weeks. Similarly, Terada and coworkers [54] reported that antibodies in urine were stable for 1 year at 2–8°C for detection of *rubella*-specific IgG antibody. Thus, once the collection and the storage procedure of urine have been established, it is practical in remote areas and mass screening compared to blood sample collection. In this study, we preserved urine samples with 0.1% NaN_3_ and kept at 4°C to avoid precipitation after thawing from frozen samples.

In conclusion, strongyloidiasis is a serious public health problem that requires early detection with highly efficient but simple diagnostic methods. The findings that diagnostic accuracy in terms of the sensitivity and specificity of the urine ELISA was comparable to serum ELISA for serodiagnostic of strongyloidiasis, indicated that urine is a promising alternative to blood sample for screening prior to confirmation by a standard diagnosis for clinical management. Since collection of urine samples is noninvasive and the handling of urine samples is much easier than that of blood, urine ELISA may be used for routine laboratory analyses, epidemiological surveillance as well as a diagnostic method in control programs for strongyloidiasis. Further studies are required to test the utility of urine ELISA for diagnosis of strongyloidiasis based on larger sample sizes in different endemic communities.

## Supporting information

S1 File A complete set of data in this study. (XLSX)
